# Bidirectional Promoter Engineering for Single Cell MicroRNA Sensors in Embryonic Stem Cells

**DOI:** 10.1371/journal.pone.0155177

**Published:** 2016-05-06

**Authors:** Hanna L. Sladitschek, Pierre A. Neveu

**Affiliations:** Cell Biology and Biophysics Unit, European Molecular Biology Laboratory, Heidelberg, Baden-Württemberg, Germany; IPMC, CNRS UMR 7275 UNS, FRANCE

## Abstract

MicroRNAs have emerged as important markers and regulators of cell identity. Precise measurements of cellular miRNA levels rely traditionally on RNA extraction and thus do not allow to follow miRNA expression dynamics at the level of single cells. Non-invasive miRNA sensors present an ideal solution but they critically depend on the performance of suitable ubiquitous promoters that reliably drive expression both in pluripotent and differentiated cell types. Here we describe the engineering of bidirectional promoters that drive the expression of precise ratiometric fluorescent miRNA sensors in single mouse embryonic stem cells (mESCs) and their differentiated derivatives. These promoters are based on combinations of the widely used CAG, EF1*α* and PGK promoters as well as the CMV and PGK enhancers. miR-142-3p, which is known to be bimodally expressed in mESCs, served as a model miRNA to gauge the precision of the sensors. The performance of the resulting miRNA sensors was assessed by flow cytometry in single stable transgenic mESCs undergoing self-renewal or differentiation. EF1*α* promoters arranged back-to-back failed to drive the robustly correlated expression of two transgenes. Back-to-back PGK promoters were shut down during mESC differentiation. However, we found that a back-to-back arrangement of CAG promoters with four CMV enhancers provided both robust expression in mESCs undergoing differentiation and the best signal-to-noise for measurement of miRNA activity in single cells among all the sensors we tested. Such a bidirectional promoter is therefore particularly well suited to study the dynamics of miRNA expression during cell fate transitions at the single cell level.

## Introduction

**Fig 1:**
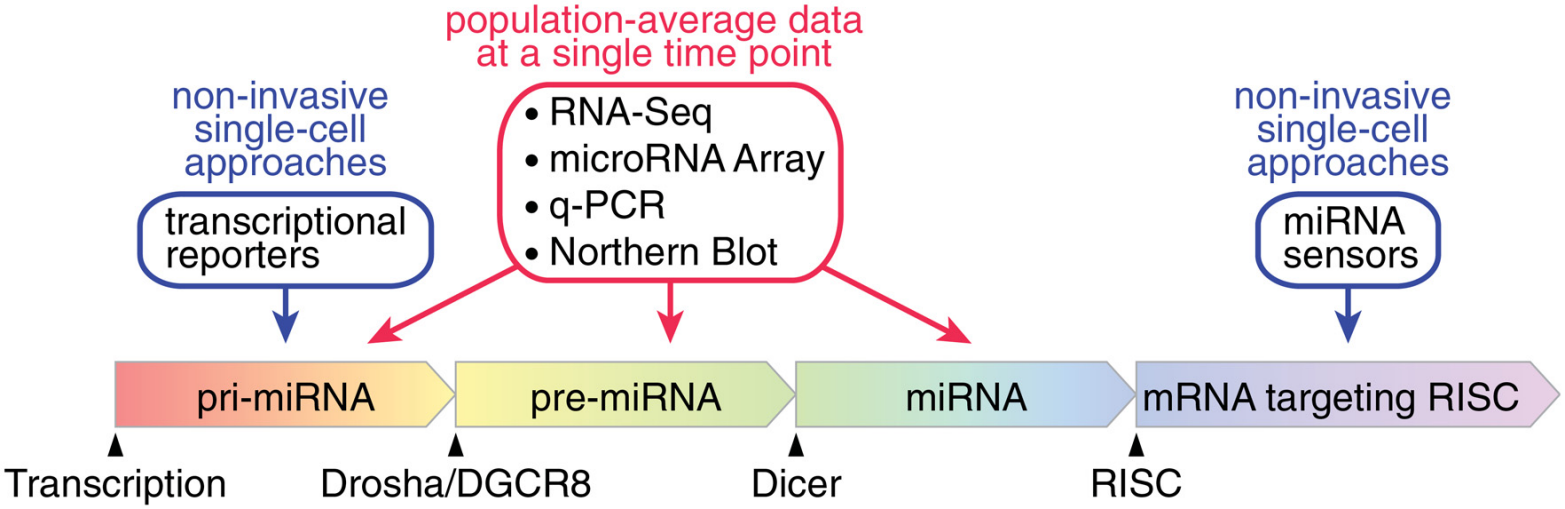
Strategies to monitor different stages of miRNA expression. miRNA expession starts with the production of primary miRNA (pri-miRNA) transcripts by RNA polymerase II or III. The microprocessor complex (Drosha/DGCR8) cleaves the flanking sequences of the miRNA-containing hairpin structure. This precursor miRNA (pre-miRNA) is then further endonucleolytically processed by Dicer to yield a double-stranded RNA fragment, whose guide strand is loaded into the RNA-induced silencing complex (RISC). This RISC complex binds to target mRNAs through partial base-pairing with the loaded miRNA and represses the expression of the bound target mRNA. Standard interrogation of cellular miRNA expression usually relies on RNA extraction procedures which typically yield population-average data. Fluorescent transcriptional reporters of miRNA genes or sensors of miRNA activity allow to measure miRNA expression and activity in single live cells.

MicroRNAs (miRNAs) are a class of small non-coding RNAs that play important roles in the post transcriptional regulation of gene expression [1]. miRNA genes are predominantly transcribed by RNA polymerase II [2] either from their own transcriptional units or being found in introns of their host genes [3–10]. The nuclear “microprocessor” complex, which is composed of the double-stranded RNA binding protein DGCR8 [11] and the catalytically active subunit RNase III Drosha [12] (Fig 1), then cleaves the primary miRNA (pri-miRNA) transcripts. The resulting hairpin structure, the precursor miRNA (pre-miRNA), is then transported to the cytoplasm [13]. Here, a complex of the RNase Dicer [14] and TRBP [15] cleaves the stem-loop pre-miRNA into 20–25 base pair long double-stranded RNA fragments. The guide strand of miRNA duplex is selectively loaded into the RNA-induced silencing complex (RISC) [16–19], where it guides RISC to target mRNAs based on sequence complementarity. Plant miRNAs pair almost perfectly with their mRNA targets and this pairing induces the cleavage of the target transcripts [20]. Only partial complementarity is needed for animal miRNAs to bind to their target genes and it renders them far more promiscuous in their target selection with single miRNAs often regulating dozens of transcripts [21]. Indeed, pairing of the “seed” region (the nucleotides 2–8 at the 5’-end of the miRNA) to the 3’-UTR of mRNAs is often sufficient for metazoan miRNAs to recognize their targets [22]. Metazoan miRNAs first elicit translational repression of their targets followed by target deadenylation and degradation [23–25].

miRNAs are predominantly found in multicellular organisms and they have been shown to play important roles in cell fate determination [26]. Not only is a functional miRNA machinery indispensable for the differentiation of embryonic stem cells [27–29], but specific miRNAs are also instrumental in directing lineage decisions [30, 31]. In fact, forced overexpression of specific subsets of miRNAs has been used to transdifferentiate somatic cells directly into other specialized cell types [32] and to reprogram somatic cells to pluripotency [33].

In addition, miRNA expression profiles are excellent classifiers of cell identities robustly distinguishing different tissues [34], cancer types [35] and stages of reprogramming [36, 37]. Most studies using miRNAs as biomarkers of cellular identity rely on data acquired by qPCR, miRNA-Seq and miRArray approaches (Fig 1). These techniques are well-established and even unbiased for miRNA-Seq but they generate population-average data from thousands of cells at single time points. Recent advances in the field of stem cell differentiation have however indicated that stem cell populations display significant heterogeneity in their differentiation potential and proclivity [38]. These developments call for non-invasive single-cell readouts of cellular identity allowing to record the cell-fate decisions and their history at the single-cell level [39].

Using a single-cell fluorescent miRNA reporter, we have recently identified a single miRNA, miR-142, as a marker distinguishing two functionally distinct states in mouse embryonic stem cells (mESCs): a state amenable to differentiation cues and a state deaf to signaling changes [40]. Importantly, these states could not be distinguished by their expression of pluripotency transcription factors, which are commonly used markers to resolve heterogeneity among ESCs [41].

Here, we discuss technical challenges and present empirically validated solutions in designing fluorescent reporters which are suitable to monitor miRNA activity in single cells during differentiation of mESCs. We designed an array of bidirectional promoters relying on three commonly used core promoters and identified a CAG-based bidirectional promoter as an ideal base for such reporters.

## Materials and Methods

### Reporter constructs

All constructs in this study were generated using the MXS-chaining strategy [42]. Briefly, an MluI-, SalI-digested MXS_*HindIII-H2B-Citrine-NheI-BglII-BamHI-bGpA* module was cloned into the MluI-, XhoI-digested SMX_DEST vector. The product of this ligation was MluI-, SalI-digested and ligated with an MluI-, SalI-digested MXS_*EF1a::HindIII-H2B-mCherry-XbaI-BglII-BclI-bGpA* module. The resulting vector was AseI-, SalI- digested and ligated with an AseI-, SalI-digested MXS_*PGK::HygroR-bGHpA* module yielding the vector in which bidirectional promoter variants were inserted.

A library of candidate bidirectional promoters was assembled by MXS-chaining into a minimal pUC19-based backbone with an EcoRV-HindIII-MluI-XhoI-SalI-HindIII multiple cloning site. Assembled bidirectional promoters were excised by HindIII-digest and ligated into the HindIII-digested, dephosphorylated vector described above. Both possible promoter orientations were kept for each promoter variant. Identity and integrity of the constructs was confirmed by analytical restriction digest.

A binding site perfectly complementary to mmu-miR-142-3p (miRBase Accession Number: MIMAT0000155) was inserted downstream of the H2B-Citrine stop codon by inserting annealed oligonucleotides (miR-142-3p_F: 5’-CTAGCAAGCTTTCCATAAAGTAGGAAACACTACAG-3’ and miR-142-3p_R: 5’-GATCCTGTAGTGTTTCCTACTTTATGGAAAGCTTG-3’) into the BamHI-, NheI-digested vector.

### Cell culture and establishment of stable mESC cell lines

Mouse ESCs E14tg2a [43] were obtained from ATCC (CRL-1821) and were maintained without feeders in “LIF+serum” medium composed of DMEM (high glucose, no glutamine, with sodium bicarbonate, Invitrogen) supplemented with 15% (v/v) ES-qualified fetal calf serum (EmbryoMax, Millipore), 10 ng/ml murine LIF (EMBL Protein Expression and Purification Core Facility), 1x non-essential amino acids, 2 mM L-glutamine, 1 mM sodium pyruvate, 100 U/ml penicillin and 100 *μ*g/ml streptomycin, 0.1 mM *β*-mercaptoethanol (all Invitrogen) on culture dishes (Nunc) coated with 0.1% (v/v) gelatin (Sigma) solution and cultured at 37°C with 5% CO_2_. Medium was changed daily and cells were passaged every other day with 0.05% Trypsin-EDTA (Invitrogen) at passaging ratios of 1/6–1/12.

Mouse ESCs E14tg2a stably expressing the fluorescent miRNA reporters were generated as described [40] and analyzed by flow cytometry. Individual clones were selected as representative cases of reporter expression if at least 75% of independently derived single-cell clones (with different transgene integration sites) displayed a comparable flow cytometry profile of reporter expression.

### Differentiation of mESCs

mESCs were seeded at 2,500 cells/cm^2^ in “LIF+serum” medium. The following day, mESCs were differentiated by withdrawing LIF from the medium.

### Flow cytometry

Cells were dissociated to single-cell suspension with 0.05% Trypsin-EDTA (Invitrogen), resuspended in D-PBS, strained through a 40 *μ*m cell strainer (BD Biosciences) and analyzed on an LSRFortessa flow cytometer (BD BioSciences). Flow cytometry data was gated on forward and side scatters using FlowJo software and further analyzed using custom Python scripts.

## Results and Discussion

### Rationale for the design of miReporters

**Fig 2:**
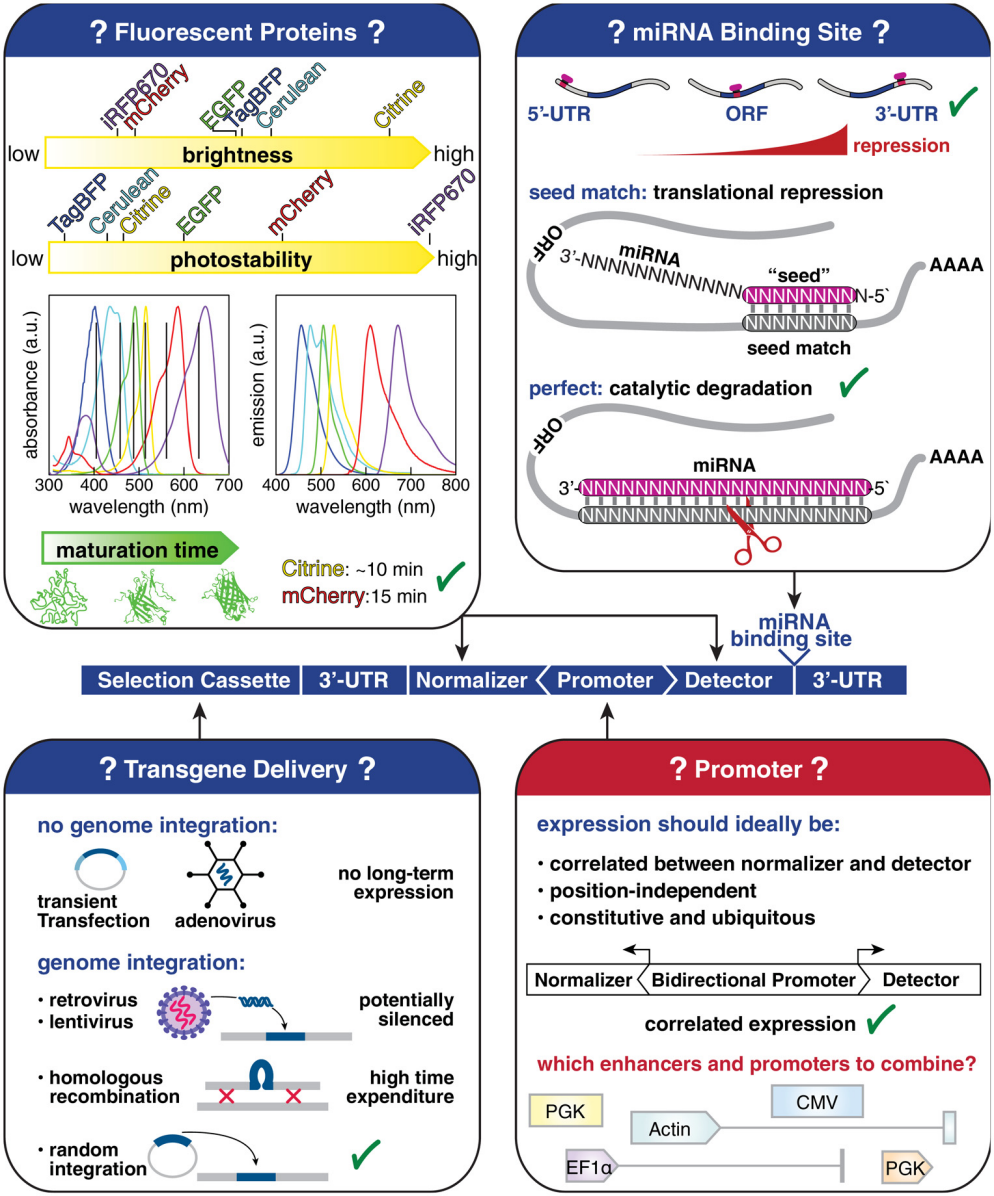
Rationale to design a miRNA reporter to read out miRNA activity in single live mESCs undergoing differentiation. The layout relies on a target site perfectly complementary to the miRNA to be monitored as perfect binding induces the catalytic cleavage of the targeted transcript but leaves the miRNA intact. Citrine and mCherry were chosen as detector of miRNA activity and normalizer because of their high brightness, high photostability, their spectral separation and fast maturation time. A bidirectional promoter layout was favored to support highly correlated expression of detector and normalizer. The generation of stable transgenic lines by random transgene integration is time-efficient and avoids the incorporation of viral sequences which might be subject to silencing. The promoter and enhancer models best-suited for the purpose of monitoring mESCs undergoing differentiation are to be determined empirically.

Pioneering miRNA activity assays relied on one or several synthetic target sites of specific miRNAs placed downstream of a sensor transgene (*β*-Galactosidase or Luciferase) [44–48]. An important consideration in the design of miRNA activity sensors is the question where to incorporate the artificial miRNA binding site (Fig 2). The vast majority of known endogenous miRNA-target interactions occurs through sites in the transcript’s 3’-UTR. Systematic analysis revealed that an artificial miRNA binding site mediated none or only marginal reporter repression when incorporated into the 5’-UTR or the ORF, but efficient repression in the 3’-UTR [49]. miRNA binding sites located near the two ends of the 3’-UTR but at least 15 nucleotides away from the stop codon tend to elicit more efficient repression than sites residing near the center [49]. Therefore, our sensor design included a multiple cloning site (MCS) to insert annealed oligonucleotides as artificial miRNA binding sites 18 nt downstream of the sensor stop codon. The rabbit *β*-Globin polyA was placed directly downstream of this MCS and served as 3’-UTR.

Most metazoan miRNAs pair imperfectly with their target mRNAs [50], a point worth considering when designing artificial miRNA binding sites. Experimental and bioinformatic analyses have been used to infer a set of rules for metazoan target recognition of which base pairing of the “seed” represents the most stringent criterion [49, 51]. Bioinformatic tools relying on these principles have been devised to predict potential miRNA target sites in mRNA transcripts [50]. However, experimental assays frequently fail to validate these predicted sites [52, 53]. The inability to accurately predict miRNA binding sites with biological function might be due to complex mRNA secondary structures, competitive binding or an incomplete understanding of the underlying physical rules controlling this process. These caveats have hindered the rational design of artificial miRNA binding sites reflecting endogenous target site properties [47]. Therefore, current miRNA sensor layouts generally favor artificial miRNA target sites which are perfectly complementary to the miRNA of interest [45, 54]. This norm is not ideal but it prevents bias introduced by the design of individual artificial miRNA binding sites. This is also essential when the designed experimental setup necessitates to compare multiple miRNA activity profiles. The approach is supported by experimental data showing that perfectly complementary miRNA binding sites prevent miRNA depletion by the sensor transcript [55]. Indeed, translational repression by partial base-pairing of the miRNA to the target mRNA necessitates at least one RISC bound per target transcript [51]. The perfect base-pairing between the miRNA and its target transcript switches the repression mechanism to the catalytic degradation of the target [55]. The miRNA remains intact after the slicing of the target mRNA the miRNA is therefore able to target other constructs. We thus decided to use sequences perfectly complementary to the miRNA of interest as binding sites (Fig 2).

The use of fluorescent proteins (FPs) instead of *β*-Galactosidase or Luciferase as sensor genes allowed to measure miRNA expression regulation down to the single cell level and circumvented enzymatic detection reactions. These advances permit non-invasive *in vivo* applications [54–56]. We decided to include a second FP as normalizer on the same construct to control for transgene expression levels and transcriptional noise. The criteria we used to select a suitable pair of fluorescent protein were high brightness, low photobleaching, sufficient spectral separation and fast maturation (Fig 2). In addition, FPs with absorbance maxima on the red-side of the spectrum were preferred in order to minimize phototoxicity. We chose Citrine (516 nm/529 nm [57]) as miRNA detector because of its superior brightness and mCherry (587 nm/610 nm [58])) as normalizer. We fused both Citrine and mCherry to histone 2B, which confers high protein stability [59] therefore maximizing signal-to-noise ratio.

Different routes to deliver miRNA sensor transgenes into cells or embryos have been previously used [47, 53, 55, 56]. Transient transfection or transduction approaches fail to ensure steady transgene expression during differentiation of mESCs, a process requiring one up to several weeks [60]. Retroviral or lentiviral systems allows for the stable integration into the host genome but often undergo silencing during ES cell differentiation [61, 62] (Fig 2). The generation of transgenic ESC lines by either homologous combination or random transgene integration allow for stable maintenance of the transgene while avoiding the incorporation of viral sequences. Gene targeting by homologous recombination is cumbersome and its efficiency is limited by the transgene size. We therefore decided to use random transgene integration for the establishment of stable miRNA reporter lines. To this end, a positive selection cassette (MXS_*PGK::HygroR-bGHpA*) was incorporated into the miRNA reporter construct (Fig 2).

The promoter driving the expression of the miRNA detector and the normalizer transcripts should critically fulfill the following criteria: i) highly correlated detector and normalizer expression ii) independence of transgene expression from the integration site iii) stable expression in mESCs and their differentiated derivatives. The first criterion can be readily implemented using a bidirectional promoter layout [47, 55, 56] (Fig 2).

The literature reports conflicting data regarding the ability of various promoters in driving stable transgene expression at high levels in pluripotent mESCs and their differentiated descendants.

The promoter of the murine 3-phosphoglycerate-kinase 1 (PGK-promoter) is the most commonly used exogenous promoter in mESCs [63, 64]. The PGK-promoter drives ubiquitous but non uniform expression in transgenic mice [65]. We have previously engineered bidirectional PGK-promoters driving the correlated expression of two transgenes in stable mESC lines and observed that the expression level can be increased by adding PGK-enhancer elements to the promoter design [42].

The human polypeptide chain elongation factor 1*α* promoter (EF1*α*-promoter) was reported to drive the robust expression of reporter genes at high levels in pluripotent ESCs and their differentiated progeny in various lineages [66–68]. However, other studies demonstrated that the EF1*α*-promoter activity is downregulated during differentiation in a cell subpopulation [69, 70]. As the analysis was performed on pools of cells instead of clonal populations [69], it is unclear if the observed silencing is a phenomenon specific to some cell types or an artifact due to the integration sites or the number of copies of the construct.

The CAG-promoter is a ubiquitous promoter composed of the immediate early CMV-enhancer, a fragment containing the promoter, first exon and intron of the chicken *β*-actin gene fused to a splice acceptor from the rabbit *β*-globin gene [71]. Transgenes can be expressed by this composite promoter in pluripotent mESCs and numerous derivatives of the three germ layers [72–75]. The CAG-promoter has been reported to drive robust transgene expression in an integration-site and copy-number independent way [70]. However, it has been found by others to be inferior in performance to the EF1*α* promoter [68].

We aimed to empirically identify a synthetic bidirectional promoter as a good candidate could not be unequivocally identified from the literature.

### Generating a library of miRNA reporters with various bidirectional promoters

**Fig 3:**
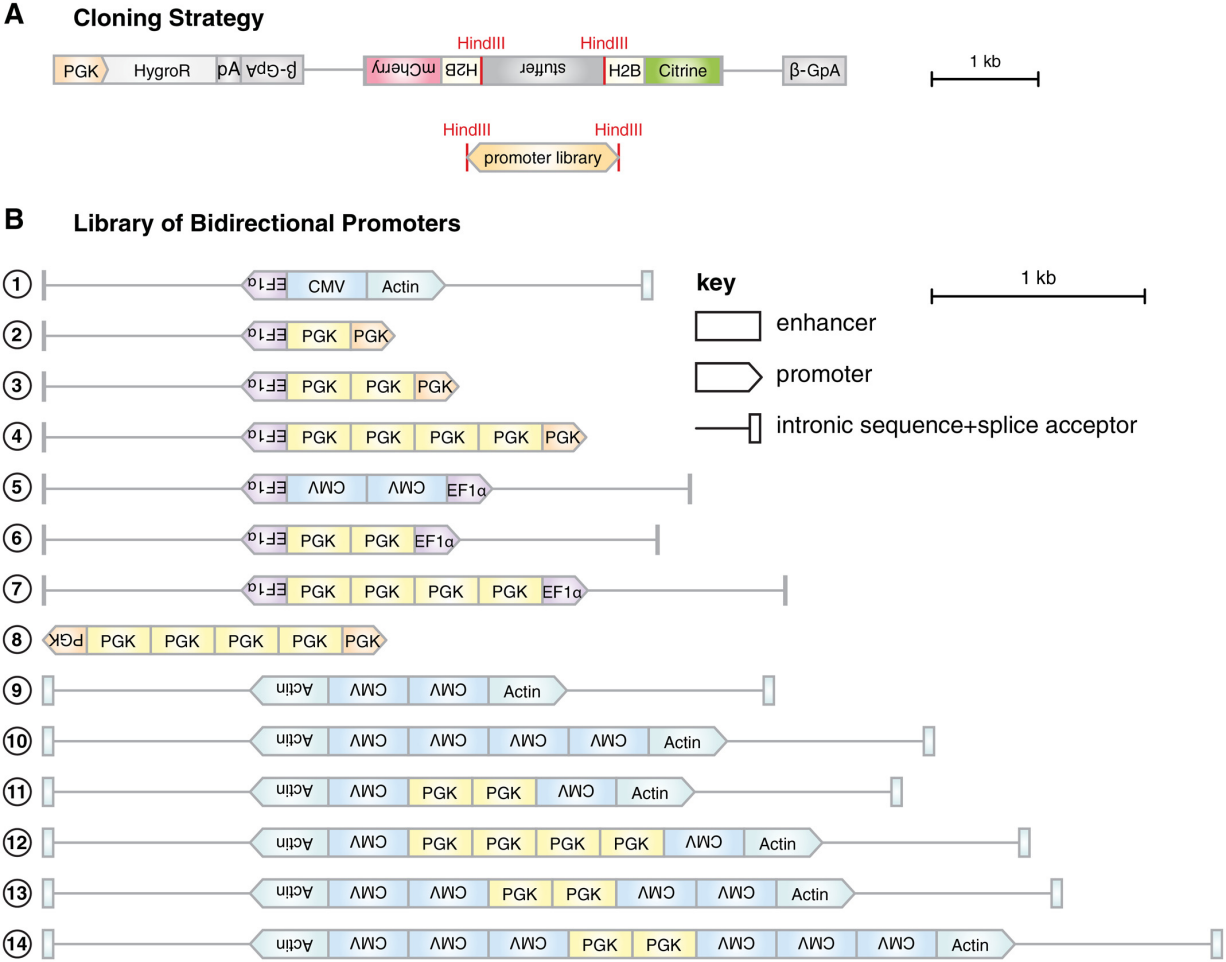
Generation of a library of miRNA reporters with various composite bidirectional promoters. (A) A library of composite bidirectional promoters was cloned non-directionally into a backbone featuring back-to-back arranged modules *H2B-Citrine-βGpA* and *H2B-mCherry-βGpA* as well as a positive selection cassette (*PGK::HygroR-bGHpA*). Scale bar: 1 kb. Annotations of modules in reverse orientation are rotated by 180°. (B) Library of composite bidirectional promoter variants. Scale bar: 1 kb. PGK: PGK-promoter (pentagon) or PGK-enhancer (rectangle), EF1*α*: EF1*α*-promoter, CMV: CMV-enhancer, Actin: the promoter, the first exon and the first intron of chicken *β*-actin gene fused to the splice acceptor of the rabbit *β*-globin gene. Annotations of modules in reverse orientation are rotated by 180°.

A library of composite bidirectional promoters was generated from the promoters and enhancers discussed above (Table 1 and Fig 3). HindIII restriction sites were inserted on both sides of each promoter variant to permit the non-directional insertion of the promoter into the vector backbone (Fig 3). This vector contained a back-to-back-arranged detector (H2B-Citrine) and normalizer (H2B-mCherry) and a positive selection cassette.

**Table 1:** List of bidirectional promoters used in the screen. See also Fig 3.

Variant	Description
①	$\overset{\leftarrow }{EF1\alpha }-{\vec{CMV}}_{Enhancer}-\vec{Actin}$
②	$\overset{\leftarrow }{EF1\alpha }-{\vec{PGK}}_{Enhancer}-{\vec{PGK}}_{min}$
③	$\overset{\leftarrow }{EF1\alpha }-\left({\vec{PGK}}_{Enhancer}\right)\times 2-{\vec{PGK}}_{min}$
④	$\overset{\leftarrow }{EF1\alpha }-\left({\vec{PGK}}_{Enhancer}\right)\times 4-{\vec{PGK}}_{min}$
⑤	$\overset{\leftarrow }{EF1\alpha }-\left({\vec{CMV}}_{Enhancer}\right)\times 2-\vec{EF1\alpha }$
⑥	$\overset{\leftarrow }{EF1\alpha }-\left({\vec{PGK}}_{Enhancer}\right)\times 2-\vec{EF1\alpha }$
⑦	$\overset{\leftarrow }{EF1\alpha }-\left({\vec{PGK}}_{Enhancer}\right)\times 4-\vec{EF1\alpha }$
⑧	${\overset{\leftarrow }{PGK}}_{min}-\left({\vec{PGK}}_{Enhancer}\right)\times 4-{\vec{PGK}}_{min}$
⑨	$\overset{\leftarrow }{AG}-\left({\overset{\leftarrow }{CMV}}_{Enhancer}\right)\times 2-\vec{Actin}$
⑩	$\overset{\leftarrow }{AG}-\left({\overset{\leftarrow }{CMV}}_{Enhancer}\right)\times 4-\vec{Actin}$
⑪	$\overset{\leftarrow }{Actin}-{\overset{\leftarrow }{CMV}}_{Enhancer}-\left({\vec{PGK}}_{Enhancer}\right)\times 2-{\overset{\leftarrow }{CMV}}_{Enhancer}-\vec{Actin}$
⑫	$\overset{\leftarrow }{Actin}-{\overset{\leftarrow }{CMV}}_{Enhancer}-\left({\vec{PGK}}_{Enhancer}\right)\times 4-{\overset{\leftarrow }{CMV}}_{Enhancer}-\vec{Actin}$
⑬	$\overset{\leftarrow }{Actin}-\left({\overset{\leftarrow }{CMV}}_{Enhancer}\right)\times 2-\left({\vec{PGK}}_{Enhancer}\right)\times 2-\left({\overset{\leftarrow }{CMV}}_{Enhancer}\right)\times 2-\vec{Actin}$
⑭	$\overset{\leftarrow }{Actin}-\left({\overset{\leftarrow }{CMV}}_{Enhancer}\right)\times 3-\left({\vec{PGK}}_{Enhancer}\right)\times 2-\left({\overset{\leftarrow }{CMV}}_{Enhancer}\right)\times 3-\vec{Actin}$

Arrows indicate the orientation of the respective modules. *PGK_min_*: minimal PGK- (mouse 3-phosphoglycerate kinase 1) promoter, *PGK_Enhancer_*: enhancer element of the PGK-promoter, *EF1α*: human EF1*α* (elongation factor 1 *α*) promoter and first intron, *CMV_Enhancer_*: CMV (cytomegalovirus) early enhancer element, *Actin*: the promoter, the first exon and the first intron of chicken *β*-actin gene fused to the splice acceptor of the rabbit *β*-globin gene.

A technical challenge is posed by the fact that inverted repeats tend to form hairpin structures, which fail to be stably propagated in bacteria [76]. We incorporated into the backbone layout a spacer element flanked by HindIII sites on either side to avoid hairpin formation between the two back-to-back arranged H2B sequences. We also avoided uninterrupted inverted repeats in the design of the bidirectional promoter variants. This principle was easily implemented owing to the fact that most enhancers function independently of orientation and distance from their target promoters [77].

### Screening of miRNA reporters to monitor miRNA activity in undifferentiated mESCs

We devised a screening process in two stages to select the bidirectional promoter reporter candidate that would be best-suited to measure miRNA activity in single mESCs undergoing differentiation. The first aim was to identify reporter variants with satisfying performance in undifferentiated mESCs under standard conditions that maintain their pluripotency (LIF+serum). Those would then be assessed in a second screen conducted under differentiation conditions by withdrawing LIF from the culture medium.

**Fig 4:**
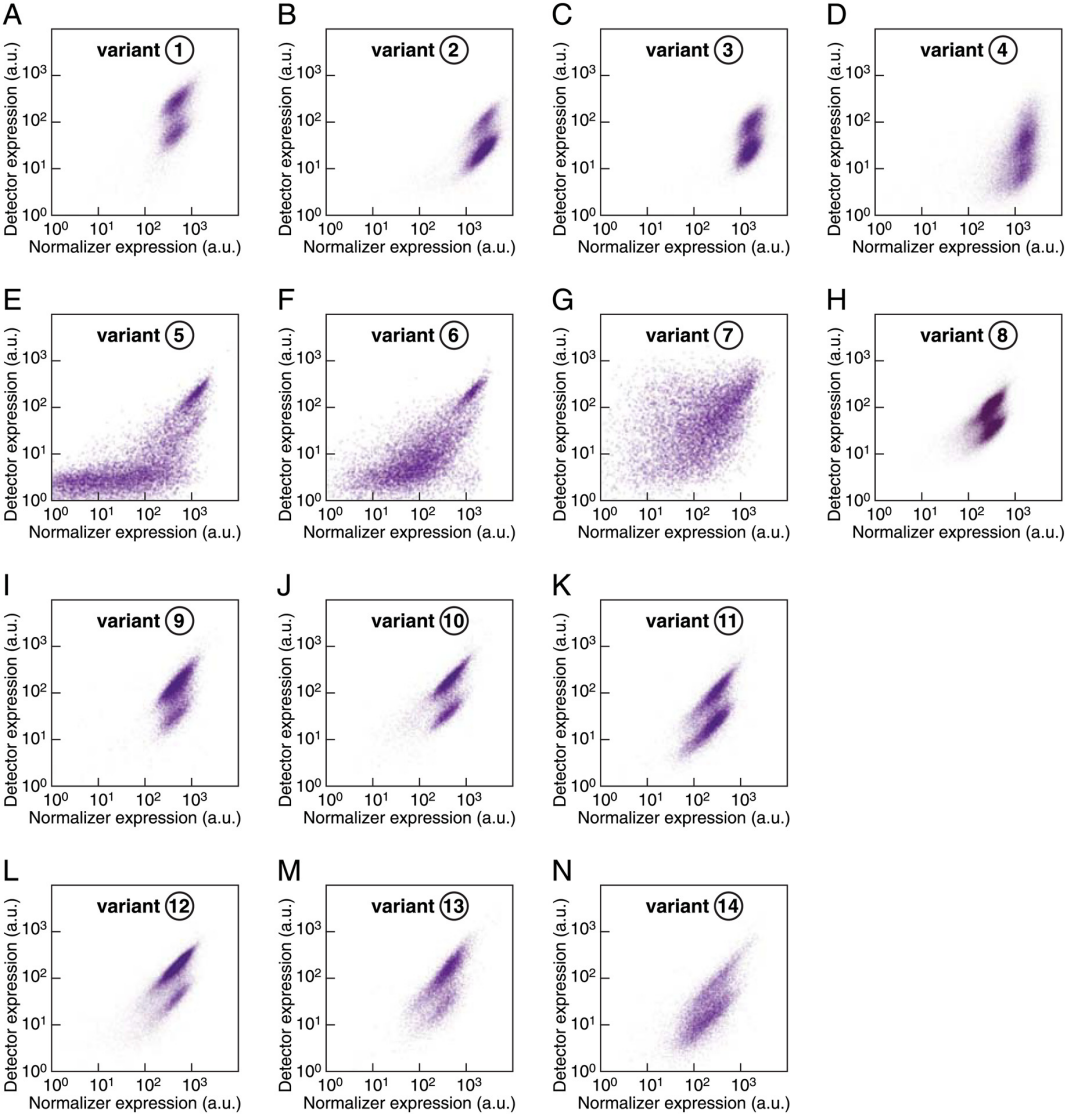
Comparing the performance of miR-142-3p activity reporters with different bidirectional promoters under pluripotency conditions. See also Fig 3. (A)-(D) Representative flow cytometry plots of clonal mESC lines expressing miR-142-3p activity reporters under the control of bidirectional promoter variants with asymmetric outer promoter elements. (E)-(H) Representative examples of clonal mESC lines expressing miR-142-3p activity reporters controlled by distinct promoters with back-to-back-arranged EF1*α*-promoters (E-G) or minimal PGK-promoters (H) as outer elements. (I)-(N) Representative examples of mESC clones expressing miR-142-3p activity reporters driven by distinct CAG-based bidirectional promoter variants.

Transcriptional noise is a critical parameter that can influence the reporter performance [78]. A first source of noise is the variability in the overall expression of the bidirectional promoter, which can be seen as an extrinsic noise. Another source of noise can affect the correlation between the expression of the two genes driven by the bidirectional promoter, which can be seen as a form of intrinsic noise. To address these points in our promoter screen, we therefore exploited the known bimodal regulation of miR-142 [40] to gauge the efficacy of a given reporter to resolve cell-to-cell variations in miRNA activity within the same population. miR-142 expression is governed by a double negative feedback loop between miR-142 itself and KRAS/ERK signaling in LIF-dependent pluripotency. This creates two stable mESC states, one with low miR-142 expression and another one with high miR-142 expression. A self-renewing mESC population will then be segregated into two subpopulations: one subpopulation with low miR-142 expression and one subpopulation with high miR-142 expression. The existence of these two subpopulations provides an internal reference to measure the performance of a given bidirectional promoter. We thus used miR-142-3p as a model miRNA, incorporating into each of the constructs variants a binding site perfectly complementary to miR-142-3p. Stable transgenic mESCs lines expressing miR-142 reporters were generated by random transgene integration. The normalizer and detector expression in single mESCs was measured by flow cytometry for each promoter candidate. Eight independent clones with distinct transgene integration sites were analyzed and representative plots are shown in Fig 4.

The comparison of the bidirectional promoter variants relying on two different core promoters (variants ①–④) revealed that a CAG-type promoter (variant ①) supported high detector expression that was not matched by the PGK-type promoters with up to four PGK-enhancer elements (variants ②–④, Fig 4A–4D). In those four cases, the EF1*α* promoter generated a high expression of the normalizer. However, back-to-back arranged EF1*α* promoter elements (variants ⑤–⑦) failed to drive robust expression despite the use of different enhancer elements (PGK- or CMV-enhancers, Fig 4E–4G). In fact, an increase of the PGK-enhancer number led to a lower performance (variants ⑥ and ⑦) and a largely uncorrelated expression of the normalizer and detector genes, making the two miR-142 states indiscernible. In contrast, four PGK-enhancers placed between two minimal PGK-promoters (variant ⑧) supported robust detector and normalizer expression (Fig 4H). Similar designs using only one or two PGK-enhancers failed to drive sufficient transgene expression (data not shown).

Finally, we compared the performance of different CAG-type bidirectional promoters with or without PGK-enhancer elements (variants ⑨–⑭, Fig 4I–4N). CAG-type bidirectional promoters generally exhibited excellent transgene expression levels and a tight correlation in detector and normalizer expression, resulting in a high resolution of the two miR-142 states. However, the transgene expression levels in individual cells were more variable for all CAG-type bidirectional promoters (variants ⑨–⑭) compared to the bidirectional PGK-promoter (variant ⑧). These variable signal intensities are not critical for flow cytometry experiments but could pose a challenge in live-imaging applications where photobleaching and phototoxicity limit laser exposure.

### Correlation between the expression of two transgenes driven by bidirectional promoter candidates

**Fig 5:**
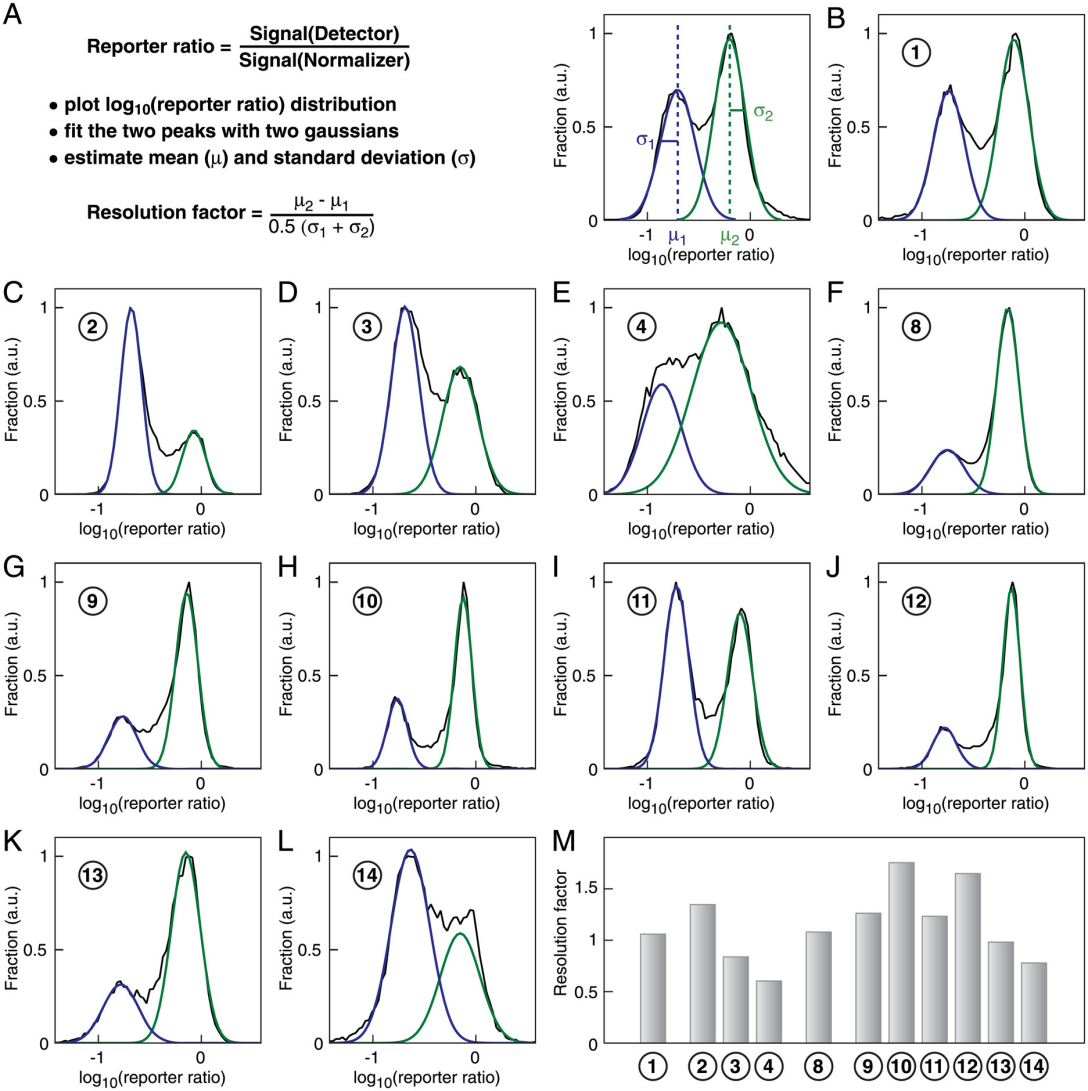
Measuring the correlation of transgene expression for different bidirectional promoters. (A) Strategy to measure the performance of bidirectional promoters at driving correlated expression of two genes. The distribution of the log-transformed reporter ratio is adjusted by a sum of two Gaussians with means *μ*_1_ and *μ*_2_ and standard deviations *σ*_1_ and *σ*_2_. The resolution factor is defined as the difference between the two means divided by the average of the standard deviations. (B)-(L) Distribution of miR-142 reporter ratio for the promoter variants ①–④ and ⑧–⑭. (M) Resolution factor for the promoter variants ①–④ and ⑧–⑭.

The performance of a miRNA activity reporter will depend on the degree of correlation between the normalizer and detector expression in single cells. miR-142 is expressed at two different levels in self-renewing mESCs, leading to a bimodal distribution of its activity reporter [40]. The log-transformed reporter ratio is well-approximated by the sum of two Gaussian distributions [40]. The resolution factor is defined as the difference between the two means divided by the average of the standard deviations of the reporter ratios for two individual states (Fig 5A). We systematically measured the resolution factor for the different candidate promoters, except for the bidirectional EF1*α* promoters (variants ⑤–⑦) that failed to drive correlated expression of normalizer and detector.

For all promoter variants, the difference between the means of the log-transformed reporter ratio for each state was similar (Fig 5B–5L). This is consistent with the fact that the ratiometric miRNA sensor is a direct measure of the cellular miRNA concentration and should not depend in theory on the promoter used to drive the construct [40]. Differences in resolution factor will thus be contributed by the width of the Gaussians, that is the degree of intrinsic noise for each bidirectional promoter variant.

The two miR-142 states were resolved in the case of asymmetric bidirectional promoters with EF1*α* driving the normalizer expression and CAG or PGK promoters (variants ① and ②) driving the detector expression (Fig 5L). An increase in the number of PGK-enhancers (variants ②–④) was accompanied by a decrease in the resolution factor (Fig 5M). CAG-based bidirectional promoters (variants ⑨–⑬) had resolution factors greater than 1 except for the variant ⑭ with two PGK- and six CMV-enhancer elements (Fig 5M). Notably, variants ⑩ and ⑫ exhibited resolution factors greater than 1.5 (Fig 5M).

### Screening of miRNA reporters to monitor miRNA activity during differentiation

**Fig 6:**
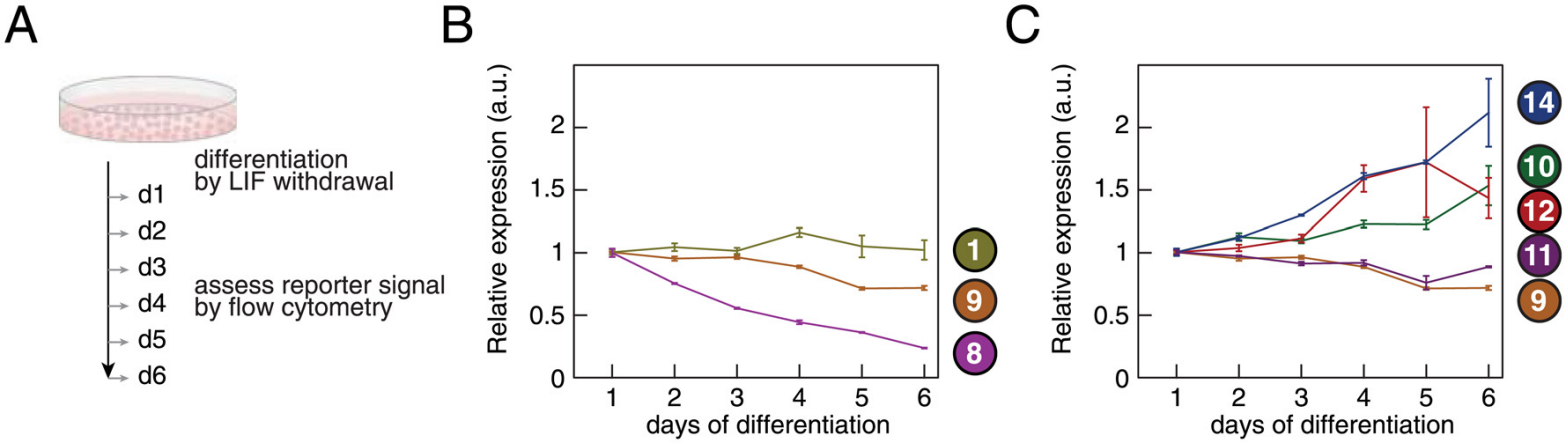
Promoter expression during mESC differentiation. (A) Experimental scheme to measure promoter activity during mESC differentiation. The normalizer expression is measured every day by flow cytometry after withdrawal from LIF. (B) Normalizer expression for promoter variants ①, ⑧ and ⑨ corresponding to EF1*α*, PGK and CAG promoters driving mCherry. Data represented as mean ± standard deviation, n = 2. (C) Normalizer expression for CAG-based promoter variants ⑨, ⑩, ⑪, ⑫ and ⑭. Data represented as mean ± standard deviation, n = 2.

After determining their expression characteristics under self-renewing conditions, we next assessed the performance of promoter variants ①, ⑧, ⑨, ⑪, ⑫ and ⑭ under differentiation conditions. To this end, we cultured the respective transgenic lines in the absence of LIF and assessed the reporter signal each day by flow cytometry (Fig 6A).

First, we examined the performance of the three core promoters EF1*α*, PGK and CAG (variants ①, ⑧ and ⑨) upon mESC differentiation. The chimeric promoter composed of an EF1*α* and a CAG-promoter element (variant ①) maintained robust reporter expression during the 6 days of differentiation (Fig 6B). In contrast, the PGK-type bidirectional promoter (variant ⑧) was gradually shut down and therefore not suited to monitor miRNA activity under differentiation conditions (Fig 6B). Finally, the CAG-based bidirectional promoter (variant ⑨) with two enhancer elements was subject to moderate downregulation (Fig 6B).

We next investigated whether additional enhancer elements could stabilize the expression of the CAG-based promoter during differentiation. Indeed, the variant with four CMV-enhancer elements (Fig 6C, variant ⑩) imparted a slight increase in reporter expression. Comparing signal evolution in cells stably expressing reporters with promoter variants ⑪, ⑫ and ⑭ added further support to the hypothesis that additional enhancer elements increased transgene expression levels as differentiation progressed.

### Conclusion

In this study, we have constructed an array of bidirectional promoters serving as the core of miRNA sensor reporter constructs. These reporters have been tested in single cells in mouse embryonic stem cells and their differentiated derivatives. Their behavior was hard to predict and had to be empirically tested for the different envisioned applications. For example, reporters relying on EF1*α* promoters combined in back-to-back orientations led to unreliable expression, precluding their utilization in single-cell measurements of miRNA activity. Similarly, sensor constructs relying on asymmetric bidirectional promoters were not suited for measurements during mESC differentiation. Indeed, we observed that differentiation affected the three popular promoters CAG, EF1*α* and PGK in distinct ways. Therefore, the correlated expression of two transgenes throughout differentiation necessitates bidirectional promoters with a symmetric design. To conclude, we found that a back-to-back CAG promoter with four CMV enhancers provided both robust expression during mESC differentiation and the best signal-to-noise of all tested reporters for measurement of miRNA activity in single cells.
